# High-Sensitive Ammonia Sensors Based on Tin Monoxide Nanoshells

**DOI:** 10.3390/nano9030388

**Published:** 2019-03-07

**Authors:** Han Wu, Zhong Ma, Zixia Lin, Haizeng Song, Shancheng Yan, Yi Shi

**Affiliations:** 1 Collaborative Innovation Center of Advanced Microstructures, Nanjing University, Nanjing 210093, China; wuhan1106@yeah.net (H.W.); mazhongnj@163.com (Z.M.); songhaizeng0501@foxmail.com (H.S.); 2 National Laboratory of Solid State Microstructures, School of Electronic Science and Engineering, Nanjing University, Nanjing 210093, China; 3 Testing center, Yangzhou University, Yangzhou 225009, China; linzixia@hotmail.com; 4 School of Geography and Biological Information, Nanjing University of Posts and Telecommunications, Nanjing 210023, China

**Keywords:** tin monoxide, nanoshell, ammonia sensor, solution method

## Abstract

Ammonia (NH_3_) is a harmful gas contaminant that is part of the nitrogen cycle in our daily lives. Therefore, highly sensitive ammonia sensors are important for environmental protection and human health. However, it is difficult to detect low concentrations of ammonia (≤50 ppm) using conventional means at room temperature. Tin monoxide (SnO), a member of IV–VI metal monoxides, has attracted much attention due to its low cost, environmental-friendly nature, and higher stability compared with other non-oxide ammonia sensing material like alkaline metal or polymer, which made this material an ideal alternative for ammonia sensor applications. In this work, we fabricated high-sensitive ammonia sensors based on self-assembly SnO nanoshells via a solution method and annealing under 300 °C for 1 h. The as fabricated sensors exhibited the response of 313%, 874%, 2757%, 3116%, and 3757% (∆G/G) under ammonia concentration of 5 ppm, 20 ppm, 50 ppm, 100 ppm, and 200 ppm, respectively. The structure of the nanoshells, which have curved shells that provide shelters for the core and also possess a large surface area, is able to absorb more ammonia molecules, leading to further improvements in the sensitivity. Further, the SnO nanoshells have higher oxygen vacancy densities compared with other metal oxide ammonia sensing materials, enabling it to have higher performance. Additionally, the selectivity of ammonia sensors is also outstanding. We hope this work will provide a reference for the study of similar structures and applications of IV–VI metal monoxides in the gas sensor field.

## 1. Introduction

Recently, ammonia (NH_3_) sensors have been widely studied by researchers and widely used in the high volume control of combustibles in the chemical industry, the control of emission of vehicles, and the monitoring of dairy products in the food industry. Currently, many nanomaterials have been utilized in ammonia sensors. These include two-dimension materials [1,2,3,4,5], IV–VI metal chalcogenides, conductive polymers, and alkali metal materials. Among these materials, owing to the low cost, high sensitivity, and environmentally-friendly nature, tin monoxide and tin dioxide have been used to fabricate ammonia gas sensors [6,7,8,9,10,11,12,13,14,15,16]. Compared with tin dioxide, tin monoxide synthesized under lower temperature is more stable and absorbs ammonia more easily when utilized as gas sensors [17,18]. 

As one of the common pollutants and toxic gases, ammonia (NH_3_) can cause several effects on the human body like irritation of the eyes, skin, throat, and respiratory system. According to the US Occupational Safety and Health Administration (OSHA), the exposure of under 35 ppm of ammonia by volume in environmental air for 15 min or under 25 ppm of volume for 8 h potentially harms people’s health [12,13]. However, it is impossible for humans to detect ammonia below 50 ppm, which reflects the importance of ammonia sensing. Hence, a highly sensitive and selective room temperature NH_3_ gas sensor is highly desirable in today′s world [19,20]. In previous work, the effect of material structure on the sensitivity of indium oxide-based ammonia sensors has been discussed [21]. Deren Yang et al. demonstrated that broken indium oxide nanotube structure with ultrahigh surface-to-volume ratio exhibited higher performance than regular nanotube, nanowire, and nanoparticle [21]. This is because the ultrahigh surface-to-volume ratio material can potentially provide larger interface to absorb gas [22]. Shell structure is another structure with ultrahigh surface-to-volume ratio that is suitable for gas sensing [23]. After referring to this work and the fabrication of CdS nanoshell structure [24], we synthesized a SnO nanoshell structure that also possesses high surface-to-volume ratio with the aim of improving the sensitivity of our ammonia sensors. SnO is a kind of monoxide and the mechanism of ammonia sensing is related to the redox reactions. As is similar to other metal oxide sensors, when the SnO is exposed to air, oxygen will be adsorbed on its surface, and oxygen molecules attract electrons. As a result, the conductivity of the SnO decreases. Then, when the sensor is exposed to a reducing gas such as NH_3_, the reducing gas may react with the adsorbed oxygen molecules and release electrons into the SnO, thereby increasing the conductivity. From this mechanism, the oxygen adsorption in the primary step is very important for the performance of the sensor [14,21]. The oxygen adsorption relies on the oxygen vacancy of the material and high oxygen vacancy density of SnO nanoshell also contributes to its high sensitivity.

In this paper, we prepared Sn_6_O_4_(OH)_4_ as precursors through a facile solution method and further prepared SnO nanoshell through different annealing conditions. The morphology, structure, and chemical composition of our samples were investigated by instruments. Among all samples annealed under different conditions, Sample 3 showed shell structure and the highest response in ammonia sensing. Compared with reported works, gas sensors prepared by Sample 3 showed a much higher response. These prepared sensors also showed outstanding selectivity and stability. The mechanism of response was revealed, and two factors that contributed to the high sensitivity of the as prepared sensor were ultrahigh surface-to-volume ratio and high oxygen vacancy density. This work provided novel structure for conductive materials which were suitable for a high performance gas sensor. We hope this work will provide new ideas for applications of IV–VI metal monoxides in the gas sensor field.

## 2. Experimental Details

### 2.1. Materials

In this experiment, all chemicals used were of analytical grade and were applied as-received, without further purification. Thioacetamide and NaOH powder were purchased from Sinopharm Chemical Reagent Co., Ltd (Shanghai, China). Stannous chloride (SnCl_2_·2H_2_O) was purchased from Aladdin Industrial Corporation (Shanghai, China). Ultrapure water that was used in the experiment was purified using the Millipore water purification system (Millipore Corporation, Burlington, MA, USA). *N*-menthylpyrrolidinone (1-methyl-2-pyrrolidinone) (NMP) was purchased from Sigma-Aldrich (St. Louis, MO, USA).

### 2.2. Synthesis Methods

In this experiment, 45 mL of deionized water and 33.75 mg of thioacetamide were introduced into a 100 mL bottle. After stirring for 1 min, 30 mg of NaOH was added to the bottle. While the solution was stirred, 101.54 mg of SnCl_2_·2H_2_O was added and the color of the solution became milky white. The reaction of

SnCl_2_·2H_2_O + H_2_O → Sn(OH)Cl + HCl (1)

took place [25]. Afterwards, the solution was stirred for another 20 min. The suspension was centrifuged for 20 min at 10,000 rpm and the precipitate was dried at 80 °C for 12 h in a vacuum oven. From our previous study, dried precipitate was Sn_6_O_4_(OH)_4_ which was a precursor of SnO nanoshell. The precursor showed shell structure and this structure could be kept only annealed under suitable conditions. In fact, we found only Sample 3 and 5 kept shell structure in subsequent tests and Sample 3 had higher density of shell than Sample 5. The dried precipitate was then annealed under different conditions. Under specific annealing conditions, the decomposition reaction of precursor

Sn_6_O_4_(OH)_4_ → 6SnO + H_2_O (2)

took place. In this work, Sample 1, 2, 3, and 4 were obtained after heating from 0 °C to 200 °C, 250 °C, 300 °C, and 350 °C for 1 h and kept at 200 °C, 250 °C, 300 °C, and 350 °C for 1 h, respectively. Sample 5 was obtained after heating from 0 °C to 350 °C for 30 min and kept at 350 °C for 30 min. In Supplementary Materials, we have added a flow chart to demonstrate the synthesis process in Figure S1. To demonstrate the difference between each sample from preparing to investigating, we concluded Table S1 in Support Information.

### 2.3. Characterization of Material

Field emission scanning electron microscopy (FESEM, JSM-7000 F, JEOL Ltd., Tokyo, Japan) was used to determine the morphology of the samples. Transmission electron microscopy (TEM) and high-resolution transmission electron microscopy (HRTEM) images were obtained using a TEM (FEI Tecnai G2 F30 S-Twin TEM, Georgia Tech, Atlanta, GA, USA) instrument. Raman spectrum was obtained using a Raman spectrometer (LabRamHR800, HORIBA, Ltd., Kyoto, Japan) that was excited by an Ar laser at 514.5 nm under 500 μW. The crystal phase properties of the samples were analyzed by a Bruker D8 Advance X-ray diffractometer (Bruker, Billerica, MA, USA) with Ni-filtered Cu Kα radiation at 40 kV and 40 mA and 2θ from 10° to 60° with a scan rate of 0.02°. X-ray photoelectron spectroscopy (XPS) analysis (PHI5000Versaprobe, ULVAC-PHI, Inc. Chigasaki, Kanagawa, Japan) was used to determine the chemical composition of the products.

### 2.4. Fabrication and Measurement of the Ammonia Sensor

**Figure 1:**
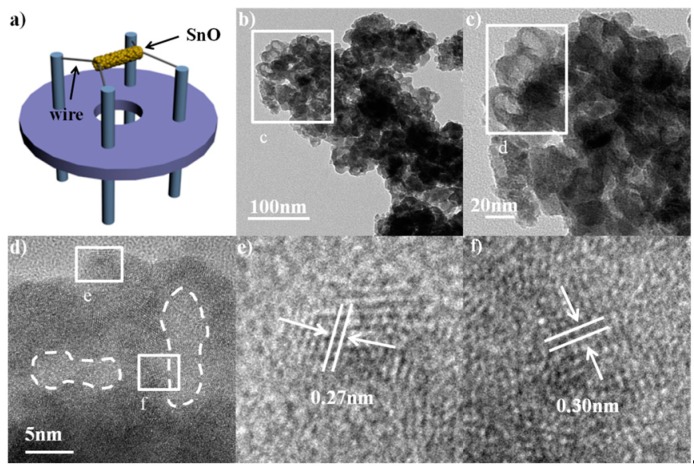
Configuration of the as fabricated devices and morphology of as prepared Sample 3. (**a**) The configuration of sensor devices. (**b**,**c**) TEM (transmission electron microscopy) image of Sample 3 at different magnifications. (**d**) TEM image of Sample 3 which expands the top-left section of (**b**). (**e**,**f**) HRTEM (high-resolution transmission electron microscopy) image of corresponding white solid frames marked in (**c**).

To fabricate ammonia sensors, 20 mg sample were mixed with 40 μL NMP (*N*-menthylpyrrolidinone 1-methyl-2-pyrrolidinone) and were grinded in a mortar for 20 min. Through a paint pen, the mixture was coated on sensors purchased from Winsen Electronic Technology Co., Ltd. (Zhengzhou, Henan Province, China). Finally, sensors were welded on substrates. The configuration of the as fabricated device is shown in Figure 1a. The as fabricated sensor devices were dried for 20 min at 50 °C in a vacuum oven. Then, ammonia gas sensing was tested by Navigation 4000 Series Smart Sensor Tester purchased from Beijing ZhongKe Micro-Nano Networking Science Technology Co., Ltd. (Beijing, China). The sensor response S was measured by systematically exposing sensors to different concentrations of ammonia (5 ppm to 200 ppm) at room temperature using the following equation S = (G_g_ − G_a_)/G_a_ × 100, while G_a_ and G_g_ are the conductance of the sample in air and ammonia gas, respectively.

## 3. Results and Discussion

### 3.1. Characteristic of SnO Nanoshell Material

In this work, Sample 3, annealed and heated from 0 °C to 300 °C for 1 h and then kept under 300 °C for 1 h, possesses the nanoshell structure and exhibits the highest performance when utilized as the conductive material of ammonia sensors. The configuration of sensor devices and morphology of the as-synthesized Sample 3 is shown in Figure 1. Figure 1a shows the configuration of the sensor devices. Figure 1b shows the TEM image of Sample 3 which exhibits the accumulation of SnO nanoshells. Figure 1c shows the higher magnification TEM image of the top-left section of Figure 1b. We clearly observed the shell structure in the top-left region of Figure 1c. Further expansion of the white solid frame in Figure 1c shows the nanoshell structure with a hollow core, presented in Figure 1d, marked with white dashed lines. The morphology of Sample 3 in Figure 1 confirmed the existence of the nanoshell structure. The HRTEM image of the regions within the white solid frames of Figure 1d were shown in Figure 1e,f, showing a lattice fringe spacing of 0.27 nm and 0.30 nm, which corresponds to the (110) plane and (101) plane of SnO, respectively [10].

**Figure 2:**
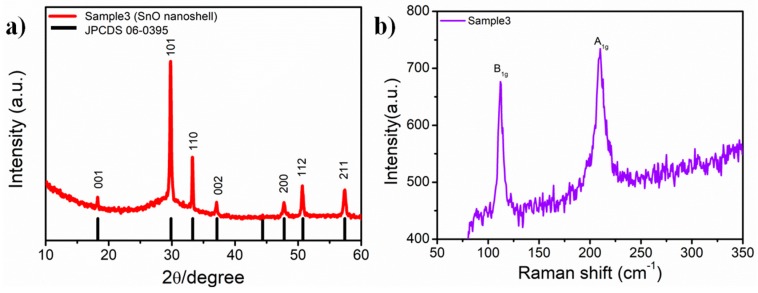
Characterization of the crystal structure of Sample 3. (**a**) X-ray diffraction of Sample 3; (**b**) Raman spectrum of Sample 3.

The crystal structure of the as-prepared Sample 3 is shown in Figure 2. Figure 2a shows the X-ray diffraction of Sample 2. Peaks at 18.2°, 29.8°, 33.2°, 37.1°, and 47.7° correspond to the (001), (101), (110), (002), and (200) crystal planes of SnO, respectively. All the diffraction peaks can be assigned to SnO (JCPDS Card No. 06-0395) [10,13]. From the X-ray diffraction, the intensity of (110) and (101) peaks was much higher than the other which matched well with these two planes and could be found easily in TEM. In addition, the Raman spectrum of Sample 3 is shown in Figure 2b. The peaks at 112 cm^−1^ and 210 cm^−1^ correspond to the B_1g_ and A_1g_ vibration mode of SnO, respectively [26]. In A_1g_ mode, Sn atoms vibrate towards or away from O atoms. B_1g_ mode corresponds to the out-of-plane vibrations of O atoms [27]. These characterizations confirmed that this material is SnO. The morphology and Raman spectra of other samples (Sample 1, Sample 2, Sample 4, and Sample 5) are provided in Figures S2–S5. From the supplementary material, Sample 3 and sample 5 showed shell structure and the density of shell structure of Sample 3 is higher than Sample 5.

**Figure 3:**
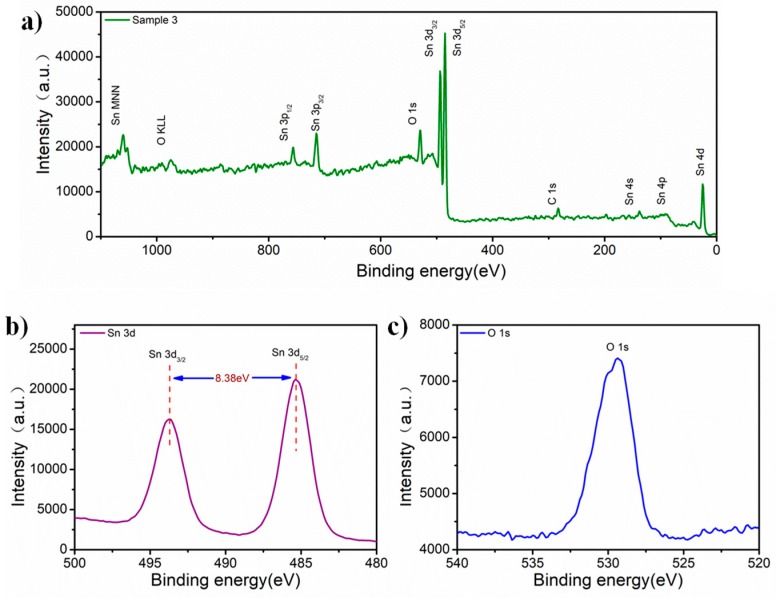
X-ray photoelectron spectroscopy of as-prepared Sample 3. (**a**) Sample 3 has Sn, O in the full spectrum; (**b**) High-resolution XPS of Sn 3d; (**c**) Sn–O bond in O 1s spectroscopy.

X-ray photoelectron spectroscopy shown in Figure 3 was used to analyze the surface chemical composition of Sample 3. As shown in Figure 3a, only peaks that correspond to Sn, O, and C were observed. Figure 3b shows the high resolution spectrum of Sn 3d. Peaks at 485.4 eV and 493.8 eV correspond to the energies of Sn 3d_5/2_ and Sn 3d_3/2_, respectively. The energy gap that reveals the amount of energy splitting between the two core levels is 8.38 eV, which corresponds to the energy splitting of Sn^2+^ [26]. Figure 3c shows the peak that corresponds to the Sn–O bond at 529.4 eV in O 1s spectroscopy. This result is consistent with previous work [28,29].

### 3.2. Test of Gas Sensor Device

**Figure 4:**
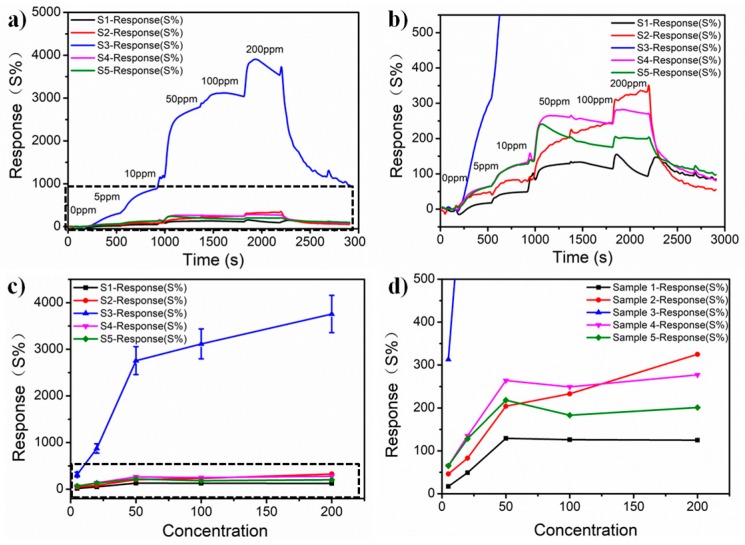
Response of ammonia gas sensors using different samples. (**a**) Response-recovery curves of the sensors up to 0–200 ppm NH_3_; (**b**) Magnification of the black dashed pane in (**a**); (**c**) Response towards five (5–200 ppm) different concentrations of NH_3_ in air; (**d**) Magnification of the black dashed pane in (**c**).

In the experiment, we prepared 15 samples (three samples for each kind) for ammonia sensing tests from 0 to 200 ppm. A sketch added in supplementary material Figure S6 was shown to describe the mechanism. The response of the fabricated ammonia gas sensors, shown in Figure 4, reveals the huge difference between Sample 3 and other samples. As shown in Figure 4a,b, the response of Sample 3 is 313%, 874%, 2757%, 3116%, and 3757% under gas concentration of 5 ppm, 20 ppm, 50 ppm, 100 ppm, and 200 ppm, respectively, which is much higher than the other four samples. This result demonstrates that the large surface area of the nanoshell structure is able to absorb more ammonia. The response to different ammonia concentrations is shown in Figure 4c,d. From Figure 4c,d, all sensors fabricated had approximate linear response to ammonia below 20 ppm. Due to saturation of absorbance to ammonia, curves have a lower slope after 50 ppm and only the response of Sample 3 and Sample 2 maintain an increasing trend. The accurate response of Sample 3 from 0 to 40 ppm was shown in Figure S7. This test aimed to reveal that the sensor fabricated with Sample 3 is sufficiently sensitive to work under lower concentrations. In supplementary material, Table S1 was also used to compare the differences of each sample. From the table, we concluded that the main factor that contributed to the highest sensitivity of Sample 3 was its highest surface to volume ratio resulting from its highest density of shell structure.

**Figure 5:**
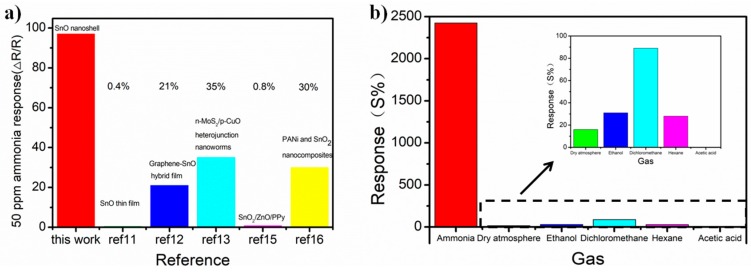
Response of as fabricated sensors compared with other work and in different gases. (**a**) Response of ammonia sensors compared with other work based on metal oxides. (The result was normalized as S = (R_a_ − R_g_)/R_a_ × 100) (**b**) Response of as fabricated sensors in different gas environments.

Response of as fabricated sensors compared with other work mentioned in this paper was shown in Figure 5a. Results in this figure were normalized as S = (R_a_ − R_g_)/R_a_ × 100 where R_a_ and R_g_ are the resistance of the sample in air and ammonia gas, respectively. From the histogram, the response of our sensor is 97%, which is much higher than the other sensing materials reported in the literature [11,12,13,15,16]. The mechanism of ammonia sensing is related to the redox reactions. When the SnO is exposed to air, oxygen will be adsorbed on its surface, and oxygen molecules attract electrons. As a result, the conductivity of the SnO decreases. Then, when the sensor is exposed to a reducing gas such as NH_3_, the reducing gas may react with the adsorbed oxygen molecules and release electrons into the SnO, thereby increasing the conductivity. During the sensing process, these reactions would take place:

O_2_ (gas) → 2O (adsorbed) (3)

O (adsorbed) + e^−^ (from SnO) → O (4)

2 NH_3_ (adsorbed) + 3 O^−^ → N_2_ + 3 H_2_O + 3e^−^ (5)

The oxygen adsorption relies on the oxygen vacancy of the material [21]. As prepared SnO is an unsaturated metal oxide that tends to absorb oxygen and be further oxidized to SnO_2_. From our previous work, the photoluminescence of oxygen vacancy on SnO nanoshell was studied which shows the high oxygen vacancy density of our SnO material [30]. In summary, the high response of our sensors depends on the high oxygen adsorption with high surface-to-volume ratio and high oxygen vacancy density of the as-prepared SnO nanoshell. Additionally, we investigated the test of selectivity of our sensors. Since there are many papers about volatile organic compound sensors based on metal oxide, we used different volatile organic compounds for comparison [31,32,33,34]. In Figure 5b, the response of our sensors in ammonia is also much higher than that of dry atmosphere or other organic gases. In the experiment, we used single gas for each test. This confirms the outstanding selectivity of our sensors. These results show the high performance of our samples. In the supplementary materials, the response (98 s) and recovery time (30 s) are shown in Figure S8. Comparison of response time and recovery time with previous work is shown in Table S2. These results show the high performance of our samples. Furthermore, we investigated the repeatability of the SnO nanoshell materials in a certain amount of 20 ppm NH_3_ and found that the SnO nanoshell material possesses good repeatability for at least one week.

## 4. Conclusions

In conclusion, we prepared SnO nanoshell through a solution method and annealing. SEM, TEM, XRD, XPS, and Raman measurements were used to characterize the present samples. The SnO nanoshell exhibited high responses of 313%, 874%, 2757%, 3116%, and 3757% under gas concentrations of 5 ppm, 20 ppm, 50 ppm, 100 ppm, and 200 ppm, respectively. The mechanism of ammonia sensing is related to the redox reactions. From the mechanism, we realized high sensitivity was due to a large surface area and higher oxygen vacancy of the SnO nanoshell. This material also showed good selectivity and repeatability in ammonia sensing. This work can potentially aid in the study of similar structures and applications of IV–VI metal monoxides for real field applications.

## Supplementary Materials

The following are available online at https://www.mdpi.com/2079-4991/9/3/388/s1, Figure S1: schematic figure about the synthesis process of tin mono oxide, Figure S2: characteristic of sample 1, Figure S3: characteristic of sample 2, Figure S4: characteristic of sample 4, Figure S5: characteristic of sample 5, Figure S6: schematic figure for the ammonia sensing mechanism of as-prepared sensors, Figure S7: Accurate response of ammonia gas sensor of sample 3, Figure S8: Response and recovery time of sample 3 (Tested under 20 ppm.), Table S1: Difference of samples in the work, Table S2: Comparison of response and recovery time with previous work.

## References

[1] Chhowalla M., Shin H.S., Eda G., Li L.J., Loh K.P., Zhang H. (2013). The chemistry of two-dimensional layered transition metal dichalcogenide nanosheets. Nat. Chem..

[2] Wang Y., Ou J.Z., Balendhran S., Chrimes A.F. (2013). Electrochemical control of photoluminescence in two-dimensional MoS_2_ nanoflakes. ACS Nano.

[3] Ning C., Qian L., Jin L., Yong W., Bai Y. (2017). Facile synthesis of fluorinated polydopamine/chitosan/reduced graphene oxide composite aerogel for efficient oil/water separation. Chem. Eng. J..

[4] Liu T., Yu K., Gao L., Chen H., Wang N., Hao L., Li T., He H., Guo Z. (2017). A graphene quantum dot decorated SrRuO_3_ mesoporous film as an efficient counter electrode for high-performance dye-sensitized solar cells. J. Mater. Chem. A.

[5] Choi W., Choudhary N., Han G.H., Park J., Akinwande D., Lee Y.H. (2017). Recent development of two-dimensional transition metal dichalcogenides and their applications. Mater. Today.

[6] Zhang F., Zhu J., Zhang D., Schwingenschlögl U., Alshareef H.N. (2017). Two-dimensional SnO anodes with a tunable number of atomic layers for sodium ion batteries. Nano Lett..

[7] Zhang D.Z., Liu J.J., Jiang C.X., Liu A.M., Xia B.K. (2017). Quantitative detection of formaldehyde and ammonia gas via metal oxide-modified graphene-based sensor array combining with neural network model. Sens. Actuators B Chem..

[8] Li Y., Yang J., Wang Y., Ma P., Yuan Y., Zhang J., Lin Z., Zhou L., Xin Q., Song A. (2018). Complementary integrated circuits based on p-type SnO and n-type IGZO thin-film transistors. IEEE Electron. Device Lett..

[9] Ogo Y., Hiramatsu H., Nomura K., Yanagi H., Kamiya T., Hirano M., Hosono H. (2008). p-channel thin-film transistor using p-type oxide semiconductor, SnO. Appl. Phys. Lett..

[10] Zhang Y., Ma Z., Liu D., Dou S., Ma J., Zhang M., Guo Z., Chen R., Wang S. (2017). p-Type SnO thin layers on n-type SnS_2_ nanosheets with enriched surface defects and embedded charge transfer for lithium ion batteries. J. Mater. Chem. A.

[11] Hien V.X., Lee J., Kim J., Heo Y. (2014). Structure and NH3 sensing properties of SnO thin film deposited by RF magnetron sputtering. Sens. Actuators B Chem..

[12] Kumar R., Kushwaha N., Mittal J. (2017). Superior, rapid and reversible sensing activity of graphene-SnO hybrid film for low concentration of ammonia at room temperature. Sens. Actuators B Chem..

[13] Sharma S., Kumar A., Singh N., Kaur D. (2018). Excellent room temperature ammonia gas sensing properties of n-MoS_2_/p-CuO heterojunction nanoworms. Sens. Actuators B Chem..

[14] Timmer B., Olthuis W., van den Berg A. (2005). Ammonia sensors and their applications—A review. Sens. Actuators B Chem..

[15] Lamdhade G.T., Raulkar K.B., Yawale S.S., Yawale S.P. (2015). Fabrication of multilayer SnO_2_–ZnO–PPy sensor for ammonia gas detection. Indian J. Phys..

[16] Khuspe G.D., Navale S.T., Bandgar D.K., Sakhare R.D., Chougule M.A., Patil V.B. (2014). SnO_2_ nanoparticles-modified Polyaniline Films as Highly Selective, Sensitive, Reproducible and Stable Ammonia Sensors. Electron. Mater. Lett..

[17] Pathak A., Mishra S.K., Gupta B.D. (2015). Fiber-optic ammonia sensor using Ag/SnO_2_ thin films: optimization of thickness of SnO_2_ film using electric field distribution and reaction factor. Appl. Opt..

[18] Yang F., Guo Z. (2015). Comparison of the enhanced gas sensing properties of tin dioxide samples doped with different catalytic transition elements. J. Colloid Interface Sci..

[19] Wang X., Li C., Huanga Y., Zhai H., Liu Z., Jin D. (2018). Highly sensitive and stable perylene sensor for ammonia detection: A case study of structure-property relationships. Sens. Actuators B Chem..

[20] Kumar R., Kushwaha N., Mittal J. (2016). Ammonia gas sensing activity of Sn nanoparticles film. Sens. Lett..

[21] Du N., Zhang H., Chen B., Ma X., Liu Z., Wu J., Yang D. (2007). Porous Indium Oxide nanotubes: layer-by-layer assembly on Carbon-nanotube templates and application for room-temperature NH3 gas Sensors. Adv. Mater..

[22] Joshi N., Hayasaka T., Liu Y., Liu H., Oliveira O.N., Lin L. (2018). A review on chemiresistive room temperature gas sensors based on metal oxide nanostructures, graphene and 2D transition metal dichalcogenides. Microchim. Acta.

[23] Joshi N., da Silva L.F., Jadhav H.S., Shimizu F.M., Suman P.H., M’Peko Je., Orlandi M.O., Seo J.G., Mastelaro V.R., Oliveira O.N. (2018). Yolk-shelled ZnCo_2_O_4_ microspheres: Surface properties and gas sensing application. Sens. Actuators B Chem..

[24] Yang M., Chan H., Zhao G., Bahng J.H., Zhang P., Král P., Kotov1 N.A. (2017). Self-assembly of nanoparticles into biomimetic capsid-like nanoshells. Nat. Chem..

[25] Ghosh S., Roy S. (2017). Effect of ageing on Sn_6_O_4_(OH)_4_ in aqueous medium—simultaneous production of SnO and SnO_2_ nanoparticles at room temperature. J. Sol–Gel Sci. Technol..

[26] Saji K.J., Tian K., Snure M., Tiwari A. (2016). 2D Tin Monoxide—An unexplored p-Type van der Waals semiconductor: Material characteristics and field effect transistors. Adv. Electron. Mater..

[27] Kachirayil J., Saji Y.P., Subbaiah V., Tian K., Tiwari A. (2016). P-type SnO thin films and SnO/ZnO heterostructures for all-oxide electronic and optoelectronic device applications. Thin Solid Film.

[28] Kraya L.Y., Liu G.F., He X., Koel B.E. (2016). Structures and Reactivities of Tin Oxide on Pt(111) Studied by Ambient Pressure X-ray Photoelectron Spectroscopy (APXPS). Top. Catal..

[29] Liang L.Y., Liu Z.M., Cao H.T., Pan X.Q. (2010). Microstructural, Optical, and Electrical Properties of SnO Thin Films Prepared on Quartz via a Two-Step Method. ACS Appl. Mater. Interfaces.

[30] Wu H., Zhou L., Yan S., Song H., Shi Y. (2017). Optical Properties of Tin Monoxide Nanoshells Prepared via Self-Assembly. Nanosci. Nanotechnol. Lett..

[31] Malik R., Tomer V.K., Dankwort T., Mishra Y.K., Kienle L. (2018). Cubic mesoporous Pd–WO_3_ loaded graphitic carbon nitride (g-CN) nanohybrids: highly sensitive and temperature dependent VOC sensors. J. Mater. Chem. A.

[32] Paulowicz I., Hrkac V., Kaps S., Cretu V., Lupan O., Braniste T., Duppel V., Tiginyanu I., Kienle L., Adelung R. (2015). Three-dimensional SnO_2_ nanowire networks for multifunctional applications: From high-temperature stretchable ceramics to ultraresponsive sensors. Adv. Electron. Mater..

[33] Postica V., Gröttrup J., Adelung R., Lupan O., Mishra A.K., de Leeuw N.H., Ababii N., Carreira J.F.C., Rodrigues J., Sedrine N.B. (2017). Multifunctional materials: A case study of the effects of metal doping on ZnO tetrapods with Bismuth and Tin Oxides. Adv. Funct. Mater..

[34] Mishra Y.K., Adelung R. (2018). ZnO tetrapod materials for functional applications. Mater. Today.

