# Supplementary material for: High-Sensitive Ammonia Sensors Based on Tin Monoxide Nanoshells
Source: Nanomaterials (Basel). 2019 Mar 7;9(3):388. doi: 10.3390/nano9030388 (PMC6474012; doi:10.3390/nano9030388)
Supplement: Supplementary file 1 [file nanomaterials-09-00388-s001.pdf]

nanomaterials

Supplementary Materials: High-Sensitive Ammonia Sensors Based on Tin Monoxide Nanoshells

Han Wu, Zhong Ma, Zixia Lin, Haizeng Song, Shancheng Yan and Yi Shi

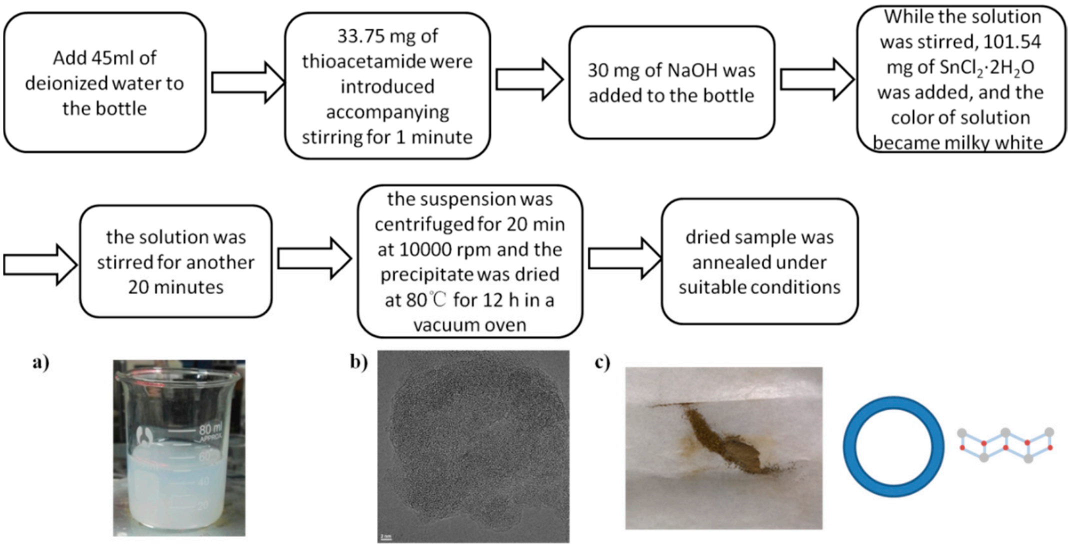

Figure S1. Schematic figure about the synthesis process of tin mono oxide. (**a**) Milky white solution after being stirred for 20 min. (**b**) TEM image of the precursor (Sn_6O_4 $(OH)_4$). (**c**) Photo of sample 3 annealed under 300 °C for 1h and structure diagram of the SnO nanoshell.

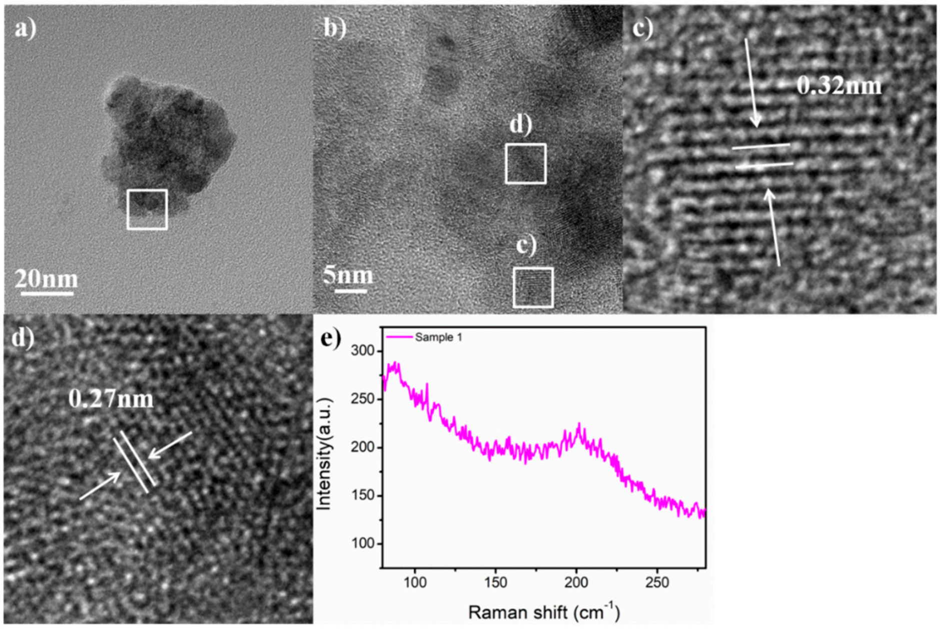

Figure S2. Characteristic of sample 1. (**a**) TEM image of sample 1. (**b**) High resolution TEM image which expands the white mark in (a). (**c**) and (**d**) Crystal lattice of sample 1 which expands the corresponding white marks in (b). (**e**) Raman spectrum of sample 1.

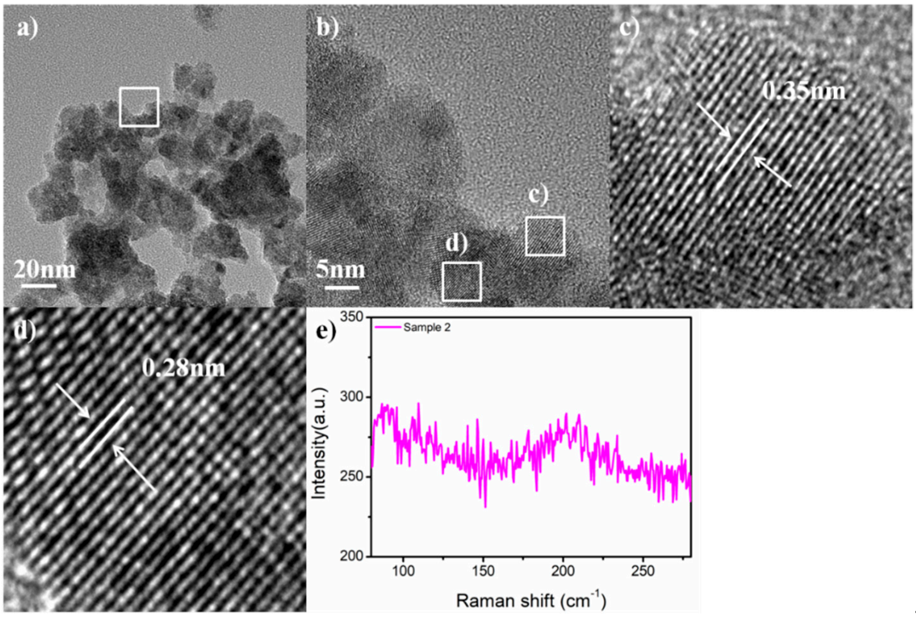

Figure S3. Characteristic of sample 2. (**a**) TEM image of sample 2. (**b**) High resolution TEM image which expands the white mark in (a). (**c**) and (**d**) Crystal lattice of sample 2 which expands the corresponding white marks in (b). (**e**) Raman spectrum of sample 2.

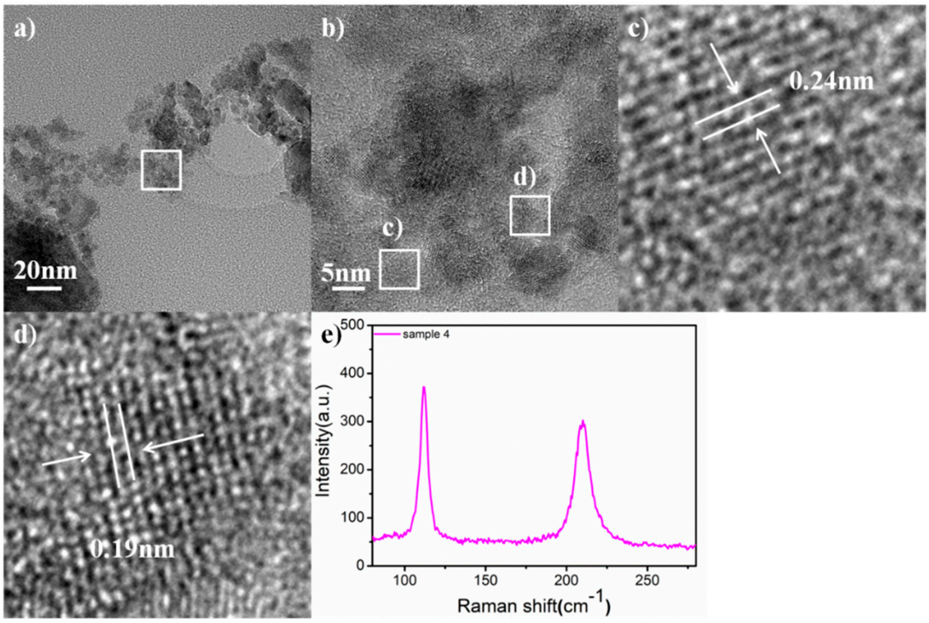

Figure S4. Characteristic of sample 4. (**a**) TEM image of sample 4. (**b**) High resolution TEM image which expands the white mark in (a). (**c**) and (**d**) Crystal lattice of sample 4 which expands the corresponding white marks in (b). (**e**) Raman spectrum of sample 4.

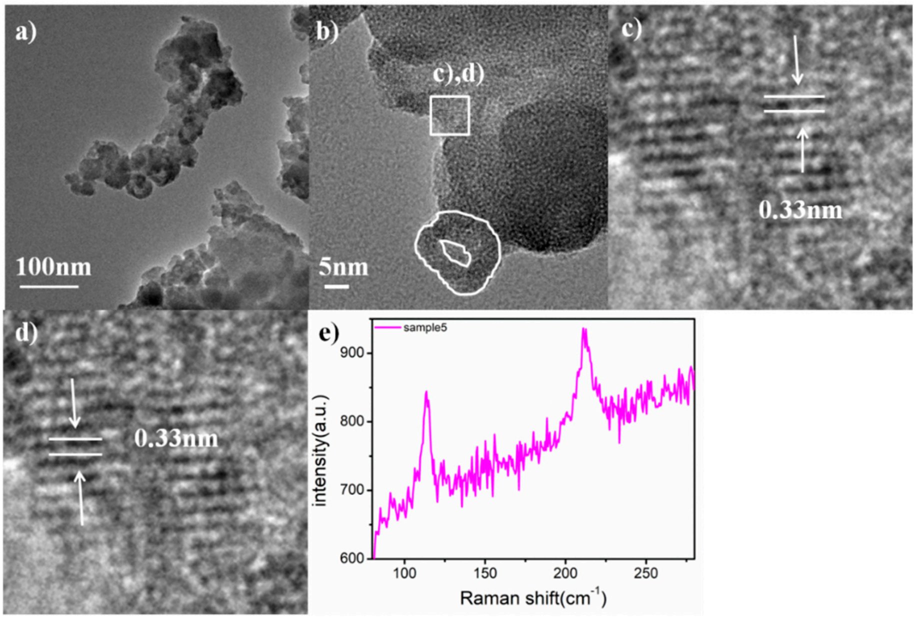

Figure S5. Characteristic of sample 5. (**a**) TEM image of sample 5. (**b**) High resolution TEM image which expands the white mark in (a). (**c**) and (**d**) Crystal lattice of sample 4 which expands the corresponding white marks in (b). (**e**) Raman spectrum of sample 4.

Figure S2–5 reveals characteristics of sample 1, 2, 4, and 5. Figure S2a–S5a and Figure S2b–S5b exhibited the TEM images and higher resolution TEM images of sample 1, 2, 4, and 5, respectively. Comparing the morphology of each sample, we found only sample 3 and sample 5 were shell structure. In Figure S2e–S5e, sample 4 and 5 together with sample 3 showed the B_{1g} and A_{1g} peaks in their Raman spectra. Additionally, the Raman spectra of sample 1 and 2 annealed under lower temperature are similar to each other and they are both different from sample 3 and 4. This consequence reminds us that precursors did not decompose totally under these conditions. As a result, the lattice fringe spacing of Figure S2c, Figure S2d, Figure S3c, and Figure S3d are 0.32 nm, 0.27 nm, 0.35 nm, and 0.28 nm, they are corresponding to (211), (212), (112), and (212) planes of $Sn_6O_4(OH)_4$. On the other hand, the lattice fringe spacing of Figure S4c, Figure S4d, Figure S5c, and Figure S5d are 0.24 nm, 0.19 nm, 0.33 nm, and 0.33 nm, they are corresponding to (002), (200), (101), and (101) planes of SnO which can be supported by their Raman spectra. This result confirms that only in higher temperatures (> 250 °C) can $Sn_6O_4(OH)_4$ be decomposed totally.

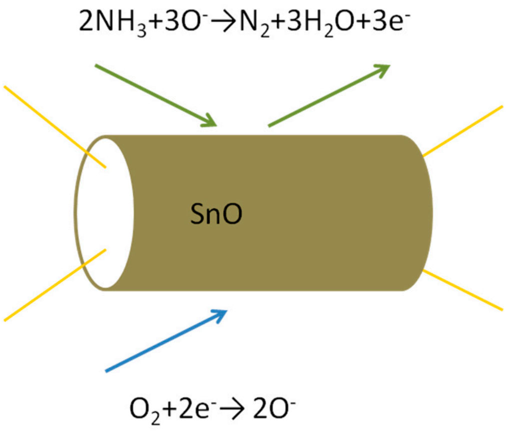

Figure S6. Schematic figure for the ammonia sensing mechanism of as-prepared sensors.

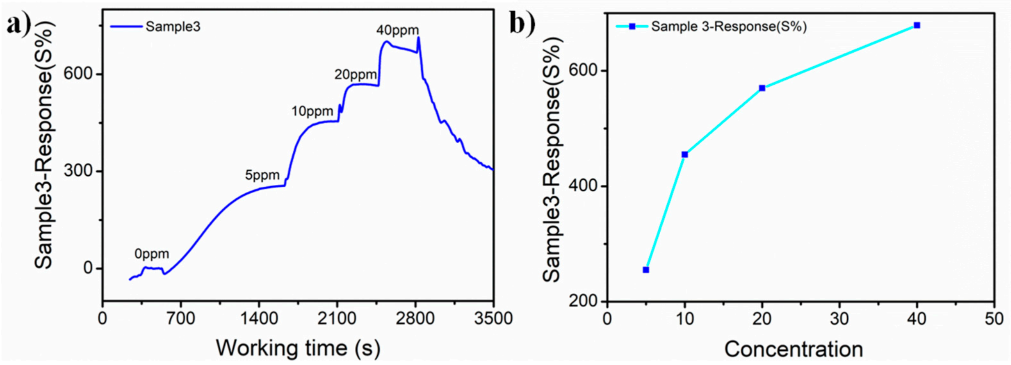

Figure S7. Accurate response of ammonia gas sensor of sample 3. (**a**) Response–recovery curves of the sensors from 0 to 40 ppm. (**b**) Response towards four (5–40 ppm) different concentrations of NH_3 in the air.

Figure S7 showed the response of the ammonia gas sensor of sample 3 from 0 to 40 ppm and the corresponding concentration-response curve. This test obviously revealed that the sensor fabricated with sample 3 is sensitive enough to work under lower concentration.

Table S1. Difference of samples in the work.

Materials	Response at 50 ppm (△G/G)	Annealing condition	Structure
Sample 1	126% ± 21%	200 °C for 1 h	
Sample 2	233% ± 24%	250 °C for 1 h	
Sample 3	2757% ± 228%	300 °C for 1 h	shell
Sample 4	249% ± 34%	350 °C for 1 h	
Sample 5	183% ± 27%	350 °C for 0.5 h	shell

Table S1 shows the difference of five different samples. The response concluded the statistic of three group experiments.

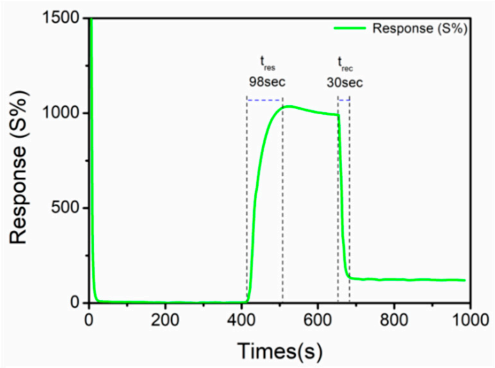

Figure S8. Response and recovery time of sample 3 (Tested under 20ppm.).

Table S2. Comparison of response and recovery time with previous work.

Sensing Material	Fabrication method	T_{res}	T_{rec}	Ref
PPy/RGO	In-situ oxidative polymerization	~200s	~90	1
SnO_2/ZnO/PPy	Electrospinning method	~67s	~106s	2
PPy/Au	Chemical oxidative polymerization	~40s	~80s	3
PPy thin films	In-situ oxidative polymerization	90s	10min	4
PANI/SnO_2	Chemical oxidative polymerization	240s	420s	5
PANI	In-situ oxidative polymerization	200s	100s	6
SnO nanoshell	Solution method	~98s	~30s	This work

Table S2 shows the response time and recovery time compared with previous work.

The response and recovery time of the ammonia sensor is shown in Figure S6 [1–6]. The response and recovery times were 98 s and 30 s, respectively. This is the common result in metal oxide ammonia sensors. Generally, strong adhesion of gas molecules to the sensing material favors sensitivity, however it also makes it more difficult to remove the sensed materials, which is the reason for the slow recovery time of 2D material-based gas sensors [7].

References

1. Sun, J.; Shu, X.; Tian, Y.; Tong, Z.; Bai, S.; Luo, R.; Li, D.; Liu, C. Facile preparation of polypyrrole-reduced graphene oxide hybrid for enhancing NH_3 sensing at room temperature. *Sens. Actuators B Chem.* **2017**, *156*, 658–664.
2. Lamdhade, G.T.; Raulkar, K.B.; Yawale, S.S.; Yawale, S.P. Fabrication of multilayer SnO_2–ZnO–PPy sensor for ammonia gas detection. *Indian J. Phys.* **2015**, *89*, 1025–1030.
3. Zhang, J.; Liu, X.; Wu, S.; Xu, H.; Cao, B. One-pot fabrication of uniform polypyrrole/Au nanocomposites and investigation for gas sensing. *Sens. Actuators B Chem.* **2013**, *186*, 695–700.
4. Joshi, A.; Gangal, S.A.; Gupta, S.K. Ammonia sensing properties of polypyrrole thin films at room temperature. *Sens. Actuators B Chem.* **2011**, *156*, 938–942.
5. Khuspe, G.D.; Navale, S.T.; Bandgar, D.K.; Sakhare, R.D.; Chougule, M.A.; Patil, V.B. SnO_2 nanoparticles-modified Polyaniline Films as Highly Selective, Sensitive, Reproducible and Stable Ammonia Sensors. *Electron. Mater. Lett.* **2014**, *10*, 191–197.
6. Kumara, L.; Rawalb, I.; Kaurb, A.; Annapoornib, S. Flexible room temperature ammonia sensor based on polyaniline. *Sens. Actuators B Chem.* **2017**, *240*, 408–416.
7. Joshi, N.; Hayasaka, T.; Liu, Y.; Liu, H.; Oliveira, O.N., Jr.; Lin, L. A review on chemiresistive room temperature gas sensors based on metal oxide nanostructures, graphene and 2D transition metal dichalcogenides. *Microchim. Acta* **2018**, *185*, 213.

© 2019 by the authors. Submitted for possible open access publication under the terms and conditions of the Creative Commons Attribution (CC BY) license (http://creativecommons.org/licenses/by/4.0/).
